# Blinded Outcome Assessment Was Infrequently Used and Poorly Reported in Open Trials

**DOI:** 10.1371/journal.pone.0131926

**Published:** 2015-06-29

**Authors:** Brennan C. Kahan, Sunita Rehal, Suzie Cro

**Affiliations:** 1 Pragmatic Clinical Trials Unit, Queen Mary University of London, London, United Kingdom; 2 MRC Clinical Trials Unit at UCL, University College London, London, United Kingdom; Harvard Medical School, UNITED STATES

## Abstract

**Objective:**

Unblinded outcome assessment can lead to biased estimates of treatment effect in randomised trials. We reviewed published trials to assess how often blinded assessment is used, and whether its use varies according to the type of outcome or assessor.

**Design and setting:**

A review of parallel group, individually randomised phase III trials published in four general medical journals *(BMJ*, *Journal of the American Medical Association*, *The Lancet*, *and New England Journal of Medicine)* in 2010.

**Main outcome measures:**

Whether assessment of the primary outcome was blinded, and whether this differed according to outcome or assessor type.

**Results:**

We identified 258 eligible trials. Of these, 106 (41%) were reported as double-blind, and 152 (59%) as partially or fully open-label (that is, they included some groups who were unblinded, such as patients, those delivering the intervention, or those in charge of medical care). Of the 152 open trials, 125 required outcome assessment. Of these 125 trials, only 26% stated that outcome assessment was blinded; 51% gave no information on whether assessment was blinded or not. Furthermore, 18% of trials did not state who performed the assessment. The choice of outcome type (e.g. instrument measured, rated, or naturally occurring event) did not appear to influence whether blinded assessment was performed (range 24-32% for the most common outcome types). However, the choice of outcome assessor did influence blinding; independent assessors were blinded much more frequently (71%) than participant (5%) or physician (24%) assessors. Despite this, open trials did not use independent assessors any more frequently than double-blind trials (17% vs. 18% respectively).

**Conclusions:**

Blinding of outcome assessors is infrequently used and poorly reported. Increased use of independent assessors could increase the frequency of blinded assessment.

## Introduction

A key component of randomised controlled trials is the assessment of patient outcomes, which involves assigning an outcome value to each trial participant. It is often recommended that outcome assessors are blinded to treatment allocation, as failure to do so can lead to systematic differences between treatment groups, resulting in biased estimates of the treatment effect [1–10]. Previous reviews have found that unblinded outcome assessment can lead to estimates of treatment effect that are exaggerated between 27% and 68%, depending on outcome type [1–3].

Despite the wide body of evidence supporting blinded outcome assessment to prevent bias in estimated treatment effects, it is still unclear how often it is used in practice. Most previous reviews have largely focused on specific disease areas [9, 11] or specific subsets of trials [12, 13], such as those that used at least some element of blinding. We therefore undertook a review of trials published in general medical journals to assess how often blinded outcome assessment was used, with a focus on trials that were not fully blinded.

## Methods

We included parallel group, individually randomized, controlled trials which were published in one of four major medical journals in 2010 (*BMJ*, *Journal of the American Medical Association*, *The Lancet*, and *New England Journal of Medicine*). Pilot and phase I or II trials, as well as articles that reported only secondary analyses were excluded. Trials were identified from the electronic table of contents for each journal. One reviewer determined whether trials met the eligibility criteria for all trials identified; a second reviewer assessed this for a subset of trials (n = 61), and agreement was 100%. Full details of the search strategy and inclusion/exclusion criteria have been published elsewhere [14]. We chose to review phase III articles from high impact general medical journals to focus on trials that were likely to have the greatest impact on patient care.

We extracted data onto a standardised form, and all trials were independently assessed by two different reviewers. Disagreements between reviewers were resolved by discussion, or by a third reviewer if necessary. For each trial, information was extracted on the blinding status of the trial, how the primary outcome was recorded, who assessed the primary outcome, and whether the assessor was blinded to treatment allocation. We identified the primary outcome as follows: (a) if only one outcome was identified as the primary, we used this; (b) if no outcomes were identified as the primary, we used the first outcome presented in the results section of the abstract; and (c) if multiple outcomes were identified as primary, we used the first of these outcomes presented in the results section of the abstract.

We classified trials as either being reported as double-blind or partially (or fully) open-label (referred to as ‘open’). We categorised trials as being ‘reported as double-blind’ if this was explicitly stated in the article, if the article stated that everyone involved in the study was blinded, or if they used a placebo or sham treatment that was described as being identical to the intervention in terms of appearance. We categorised trials as being ‘reported as open’ when it was explicitly stated, or implied through the description of the interventions, that at least some trial personnel were not blinded to treatment allocation. This included (but was not limited to) participants, those administering the intervention, those providing medical care apart from the intervention, and those assessing outcomes.

### Definition of outcome and assessor types

Outcomes were grouped into the following categories: instrument measured, rated, naturally occurring event, action-based event, all-cause mortality, other, or a composite of multiple outcome types. Full details are shown in Table 1. We defined instrument measured, rated, naturally occurring and action-based events as requiring assessment. We defined all-cause mortality as not requiring assessment. For outcomes defined as ‘other’, we decided on a case by case basis whether they required assessment.

**Table 1:** Definition of outcome types.

Outcome type	Definition	Assessor	Examples
Instrument measured	Measurements that are directly observed from an instrument, without requiring interpretation of the output by an assessor.	The person who operated the instrument which directly provided outcome results.	The patient’s blood pressure at 6 months; the assessor is the person who takes the patient’s blood pressure.
Rated	A score or summary measure that is assigned to some aspect of the patient’s wellbeing.	The person who makes the rating.	The patient completes a visual analogue scale from 0-100mm indicating how breathless they are; the assessor is the patient.
Naturally occurring event	An event that is not dependent on a direct action taken by a physician, carer, or the patient (i.e. they occur naturally), and requires interpretation of whether the event occurred or not by an assessor.	The person who determines whether the event occurred or not.	Myocardial infarction; the assessor is the person who judges whether the event occurred.
Action-based event	An event that occurs as a direct result of an action taken.	The person who made the decision which led to the event.	Whether the patient undergoes surgery to alleviate symptoms during follow-up; the assessor is the one who decided the patient required surgery.
All-cause mortality	The occurrence of death from any cause.	Not required.	Mortality from any cause within 90 days of randomisation.
Other	Any outcome that does not fall into any of the above definitions.	Dependent upon outcome.	The distance the patient is able to walk during a shuttle-walk test.
Composite of multiple outcome types	An outcome for which two or more of the above definitions apply.	Dependent on which of the above components are included in the outcome definition.	A composite of either death from uncontrolled bleeding, or requirement for surgery to control bleeding; the assessors are the person who determines whether mortality was from uncontrolled bleeding, and the person who determined whether the patient required surgery.

Assessors were classified as follows: participant, carer or physician, and independent assessors. Carers or physicians were people who provided some aspect of medical care, or helped to deliver the intervention. Independent assessors were those who, apart from assessing the outcome, had no other involvement with participants. For each outcome, we defined the assessor as the person who primarily recorded or judged the outcome. For example, for rated outcomes, the assessor was the person who made the rating; for naturally occurring events, the assessor was the person who decided whether the event had occurred or not; for action-based events, the assessor was the person who made the decision which led to the event; and for instrument measured outcomes, the assessor was the person who operated the instrument which directly provided outcome results.

We defined outcome assessment as being blinded if the article either (a) stated that blinded outcome assessment had been performed; or (b) identified who assessed the outcome, and identified this person as being blinded. We defined outcome assessment as being unblinded if the article either (i) stated that the assessment was not blinded; or (ii) identified the person who performed the assessment, and identified this person as being unblinded. When the assessment did not fall into either category, we listed it as unclear.

## Results

### Trial characteristics

In total, 258 trials met our eligibility criteria and were included in our review. General trial characteristics are shown in Table 2. Overall, 106 trials (41%) were reported as double-blinded, and 152 (59%) as partially or fully open-label (hereafter referred to as ‘open’). Of the trials reported as double blind, the majority used a pharmacological intervention (n = 97/106, 92%). In comparison, the majority of open trials used a non-pharmacological intervention (n = 100/152, 66%). Open trials often had poor descriptions of who was blinded; 61% did not state whether participants were blinded, and 68% did not state whether carers or physicians were blinded.

**Table 2:** Characteristics of included trials.

	Reported as double-blind (n = 106)	Reported as partially or fully open-label* (n = 152)
Intervention type—no. (%)		
Pharmacological	97 (92)	52 (34)
Other	9 (8)	100 (66)
Participants blinded—no. (%)		
Blinded	54 (51)	11 (7)
Unblinded	0 (0)	47 (31)
Not stated	52 (49)	93 (61)
NA	0 (0)	1 (1)
Carers or physicians blinded—no. (%)		
Blinded	42 (40)	5 (3)
Unblinded	1 (1)	40 (26)
Not stated	62 (58)	103 (68)
NA	1 (1)	4 (3)
Outcome type—no. (%)		
Instrument measured	29 (27)	27 (18)
Rated	18 (17)	25 (16)
By participant	8	17
By other	10	8
Naturally occurring event	30 (28)	28 (18)
Action based event	2 (2)	9 (6)
Decision by participant	1	3
Decision by other	1	6
All-cause mortality	6 (6)	26 (17)
Other	4 (4)	2 (1)
Composite of multiple outcome types	17 (16)	35 (23)
Number of outcome measures for trials with a composite outcome—no.		
2	9	25
3	5	8
4	3	1
5	0	1

*This includes any trial in which some groups were unblinded, including (but not limited to) patients, those delivering the intervention, or those in charge of medical care.

### Use of blinded outcome assessment

**Fig 1:**
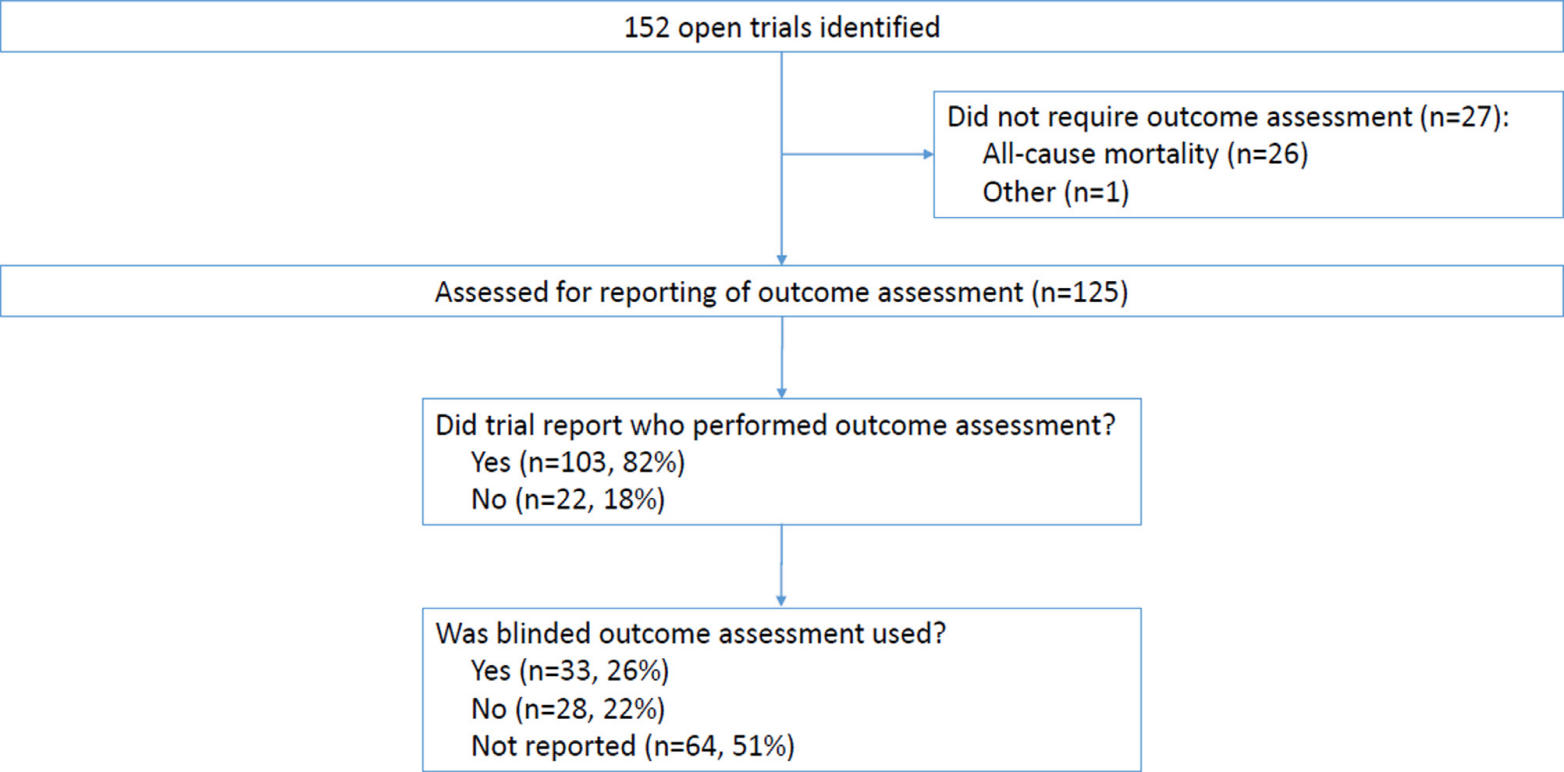
Results for open trials.

Results are shown in Table 3 and in Fig 1. In total, 27 open trials used an outcome that did not require formal assessment (26 all-cause mortality, 1 other). Among the 125 open trials where outcome assessment was required, only 33 (26%) stated that they used blinded outcome assessment; 64 (51%) did not report whether assessment was blinded or unblinded. Furthermore, 22 trials (18%) did not state who performed the assessment.

**Table 3:** Assessment of the primary outcome. *

	Reported as double-blind (n = 100)	Reported as partially or fully open-label** (n = 125)
Who assessed the primary outcome—no. (%)		
Participant	11 (11)	22 (18)
Carer or physician	33 (33)	25 (20)
Independently assessed	18 (18)	21 (17)
Not stated	23 (23)	22 (18)
Assessed by multiple groups	15 (15)	35 (28)
Number of assessment groups used for outcomes assessed by multiple parties—no. (%)		
2	10	34
3	4	1
4	1	0
Was outcome assessment blinded? (all trials) —no. (%)		
Blinded	46 (46)	33 (26)
Unblinded	0 (0)	28 (22)
Not stated	54 (54)	64 (51)

*This table only includes trials which required assessment

**This includes any trial in which some groups were unblinded, including (but not limited to) patients, those delivering the intervention, or those in charge of medical care.

### Blinding status by outcome or assessor type

Results are shown in Tables 4 and 5. In open trials, the choice of outcome type appeared to have little effect on whether blinded outcome assessment was used. The proportion of trials using blinded assessment for instrument measured outcomes, rated outcomes, naturally occurring events, or composite outcomes varied between 24–32%. The one exception was action-based events, where only one open trial (11%) used blinded assessment.

**Table 4:** Blinding of different outcome types in trials reported as partially or fully open-label.

Instrument measured outcomes—no. (%)	
Blinded	8/27 (30)
Unblinded	4/27 (15)
Not stated	15/27 (56)
Rated outcomes—no. (%)	
Blinded	6/25 (24)
Unblinded	5/25 (20)
Not stated	14/25 (56)
Naturally occurring events—no. (%)	
Blinded	9/28 (32)
Unblinded	5/28 (18)
Not stated	14/28 (50)
Action-based events—no. (%)	
Blinded	1/9 (11)
Unblinded	6/9 (67)
Not stated	2/9 (22)
Multiple outcome types—no. (%)	
Blinded	9/35 (26)
Unblinded	8/35 (23)
Not stated	18/35 (51)

**Table 5:** Blinding status by assessor type in trials reported as partially or fully open-label.

Participant—no. (%)	
Blinded	1/22 (5)
Unblinded	9/22 (41)
Not stated	12/22 (55)
Carer or physician—no. (%)	
Blinded	6/25 (24)
Unblinded	7/25 (28)
Not stated	12/25 (48)
Independently assessed—no. (%)	
Blinded	15/21 (71)
Unblinded	1/21 (5)
Not stated	5/21 (24)
Unclear who assessed—no. (%)	
Blinded	2/22 (9)
Unblinded	2/22 (9)
Not stated	18/22 (92)
Assessed in multiple ways—no. (%)	
Blinded	9/35 (26)
Unblinded	9/35 (26)
Not stated	17/35 (49)

Conversely, the rates of blinded outcome assessment varied substantially depending on the choice of assessor. Only one open trial (5%) which used patient assessment and 6 trials (24%) which used a carer or physician assessment used blinded assessment; in comparison, 15 trials (71%) using an independent assessor used blinded assessment.

There did not appear to be any difference between double-blind and open trials in the use of independent assessors (double-blind 18% vs. open 17%). However, there may have been differences across specific outcomes; for example, open trials used independent assessment for 43% of naturally occurring events, compared with 27% of double-blind trials.

## Discussion

There is substantial evidence to suggest that unblinded outcome assessment can lead to biased estimates of treatment effect. It is therefore recommended that blinded outcome assessment is used to avoid this source of bias. Our review identified 125 partially or fully open-label trials which required outcome assessment. Despite recommended practice, only 26% of trials used blinded assessment. The true figure may be higher, but is difficult to ascertain for certain due to poor reporting; over half of trials did not provide any information on whether outcome assessors were blind or not. Furthermore, 18% of trials did not state who performed the assessment.

We found that lack of blinded assessment was not associated with the outcome type (apart from action-based outcomes), but did differ according to the assessor. Assessment was blinded in only 5% of trials using patient assessment, and 24% using physician assessment. In contrast, assessment was blinded in 71% of trials using an independent assessor.

Despite the fact that using an independent assessor can substantially increase the feasibility of using blinded assessment, and thus reduce the potential for bias, we found that open trials were no more likely than double-blinded trials to use independent assessment. This surprising result has been noted before by *Dechartes et al* [15], who found that adjudication committees (typically a committee consisting of clinical experts not involved in patient care) were equally likely to be used in trials of low and high risk of ascertainment bias.

Overall, reporting of various aspects of blinding in open trials was poor. Very few trials explicitly stated whether patients or carers were blinded. This is similar to what has been found in previous studies [13, 16], and suggests that guidelines for better reporting have not been as well adopted as they should be. Given that randomised trials are often used in treatment guidelines, it is essential that the methodology of these trials is clearly reported so that others can adequately judge the relative merits of each trial.

Problematic reporting was not limited to open trials. Amongst self-reported double-blind trials, only 49% reported whether participants were blinded, 58% whether carers or physicians are blinded, and 54% whether outcome assessment was blinded. Previous research has found that some self-reported double-blind trials are actually partially open [13], and we found one trial which described itself as double-blind but actually had unblinded carers or physicians. Given the lack of details surrounding blinding in our review, it is possible that a number of other self-reported double-blind trials were actually open. It is therefore important even for double-blind trials to carefully describe who was (or was not) blinded.

Only 9 of 109 non-pharmacological trials (8%) used a double-blind design. This is not surprising given the challenges associated with blinding patients and physicians in these scenarios [17]. However, useful methods of blinding in these circumstances have previously been documented by *Boutron et al* [12], and could be more frequently adopted.

It is unclear why so few open trials used blinded outcome assessment. In some circumstances, blinded assessment is not feasible, for example patient reported outcomes in trials when patients are unblinded due to the nature of the intervention. In some cases when blinded outcome assessment is not possible, it may be possible to modify the outcome definition to reduce the risk of bias [18]. However, in many cases blinded assessment is possible [11, 12, 19]. For example, in most trials it is possible to have an independent person who is not otherwise involved in the medical care for a specific patient (and is therefore blinded to their treatment allocation) to assess their wellbeing. Often, photos or recordings can be taken and sent to an independent adjudication committee for assessment, and with new technological developments, this is a particularly promising way of ensuring blinded assessment.

The use of independent assessors may also be helpful in double-blind trials where the integrity of the blind is uncertain. For example, in some double-blind trials, the methods used to blind participants and investigators may fail, and they may become aware of the treatment allocation. Conversely, patients or physicians may be able to guess their treatment allocation based on their symptoms or side-effects. It is unclear how often this inadvertent unblinding occurs in practice, as it is rarely tested or reported [20, 21]. Therefore, more frequent use of independent assessors could also be considered in double-blind trials where the integrity of the blind is uncertain.

There are some limitations to our study. The articles we reviewed were written prior to the release of the 2010 CONSORT statement [22], and reporting may have subsequently improved since then. Secondly, we included only trials from high impact general medical journals; results may not be generalizable to lower impact or specialist journals.

## Conclusions

Blinded outcome assessment was poorly reported, and was infrequently used. Investigators should consider using independent assessors more often to increase the feasibility of blinded assessment.
